# Searching for the Transcriptomic Signature of Immune Tolerance Induction—Biomarkers of Safety and Functionality for Tolerogenic Dendritic Cells and Regulatory Macrophages

**DOI:** 10.3389/fimmu.2018.02062

**Published:** 2018-09-19

**Authors:** Juan Navarro-Barriuso, María José Mansilla, Eva M. Martínez-Cáceres

**Affiliations:** ^1^Division of Immunology, Germans Trias i Pujol University Hospital and Research Institute, Barcelona, Spain; ^2^Department of Cellular Biology, Physiology and Immunology, Universitat Autònoma de Barcelona, Barcelona, Spain

**Keywords:** biomarkers, tolerogenic dendritic cells, regulatory macrophages, tolerance mechanisms, genetic markers, immune tolerance, regulatory dendritic cells

## Abstract

The last years have witnessed a breakthrough in the development of cell-based tolerance-inducing cell therapies for the treatment of autoimmune diseases and solid-organ transplantation. Indeed, the use of tolerogenic dendritic cells (tolDC) and regulatory macrophages (Mreg) is currently being tested in Phase I and Phase II clinical trials worldwide, with the aim of finding an effective therapy able to abrogate the inflammatory processes causing these pathologies without compromising the protective immunity of the patients. However, there exists a wide variety of different protocols to generate human tolDC and Mreg and, consequently, the characteristics of each product are heterogeneous. For this reason, the identification of biomarkers able to define their functionality (tolerogenicity) is of great relevance, on the one hand, to guarantee the safety of tolDC and Mreg before administration and, on the other hand, to compare the results between different cell products and laboratories. In this article, we perform an exhaustive review of protocols generating human tolDC and Mreg in the literature, aiming to elucidate if there are any common transcriptomic signature or potential biomarkers of tolerogenicity among the different approaches. However, and although several effectors seem to be induced in common in some of the most reported protocols to generate both tolDC or Mreg, the transcriptomic profile of these cellular products strongly varies depending on the approach used to generate them.

## Introduction

The immune system develops complex and sophisticated reactions, which are able to differentiate between what is dangerous and what is innocuous for the host (1), thus specifically attacking pathogens and other potentially dangerous antigens while remaining unresponsive against whether non-dangerous or self-molecules. This balance between immunogenicity and tolerance is orchestrated in the periphery by professional antigen-presenting cells (APC), such as dendritic cells (DC) and, in a lesser extent, macrophages, which direct the immune response depending on the characteristics of the antigen and the cytokine milieu they encounter (2). Briefly, DC are in charge of both the initiation of the adaptive immune response and the control or abrogation of the inflammatory processes once the immunogenic antigen has been cleared. For this regulatory role, DC can deploy several mechanisms such as the induction of anergy or deletion of the activated immune cells, as well as, the activation of regulatory T cells (Treg) in an antigen-specific manner. Therefore, since DC have the potential to both stimulate or inhibit immune responses, the role of these cells in the immune system is complex and bidirectional (3–6). By their part, macrophages also play a minor role as APC, developing some of the regulatory processes mentioned above, although their main function consists in the clearance of cell debris, pathogens and other molecules after the immune response has concluded (7).

Eventually, the immune homeostasis can be disturbed due to a malfunction of the immune system, thus setting up immunogenic responses toward self-antigens from specific tissues and organs, which may lead to the development of autoimmune diseases. In the last years, there has been a significant progress in the knowledge of the mechanisms of immune regulation mediated by APC. Consequently, the development of novel autologous cell therapies capable of re-educating the immune system toward a tolerogenic profile has been postulated as a promising therapeutic alternative to conventional, unspecific immunomodulatory and immunosuppresive drugs, which often present severe side effects and a relatively poor efficacy (8).

So far, a wide variety of *in vitro* protocols has been established for the generation of immune tolerance-inducing DC—or tolerogenic DC (tolDC)—and regulatory macrophages (Mreg). Moreover, some of these cell products have been successfully translated from the bench to the bedside in the last few years, being tested in Phase I clinical trials in patients with autoimmune diseases—such as type 1 diabetes, rheumatoid arthritis or Crohn's disease—as well as, kidney transplantation, demonstrating in all cases that tolerogenic cell therapies are safe and well tolerated, without relevant side effects (9–13). In addition, many other studies are currently ongoing (14). These results, therefore, support the use of tolDC and Mreg as novel and safe approaches aiming to restore the immune tolerance. However, given the wide variety of protocols available for the generation of these cell products, finding objective and measurable biomarkers to characterize tolDC and Mreg and compare their characteristics between different approaches and laboratories remains one of the main obstacles to overcome.

In this context, the identification of differentially expressed (up- or down-modulated) genes in tolDC and/or Mreg constitutes one of the best tools for the definition of biomarkers of tolerogenicity, since they can provide more robust and reliable information compared to conventional methods such as phenotypical characterization by flow cytometry (with high variability) or functional studies (which require several days), as it will be further discussed below. In the case of tolDC and Mreg, these biomarkers would be able to guarantee the proper generation of the therapeutic cell product, ensuring that the cells are both safe and tolerogenic. Therefore, the ideal biomarker would be one that is selectively overexpressed or repressed in the tolerance-inducing cell product compared to its respective mature immunogenic steady-state control condition.

With that purpose, here we review the main human tolDC- and Mreg-inducing protocols reported on the literature. We specifically focus on the different agents and drugs used to generate these cell products, in order to define a catalog of genes and/or proteins induced by these stimuli and thus try to find potential and universal biomarkers of tolDC and Mreg.

## Tolerogenic dendritic cells as key tolerance-inducing players and their transcriptomic signature

DC constitute an heterogeneous subset that includes classical, plasmacytoid, and monocyte-derived myeloid DC (15). In their immature state (iDC), DC are mainly antigen-capturing cells with tolerance-inducing functionality. However, once in the presence of a pro-inflammatory stimulus such as TNF-α, lipopolysaccharide (LPS) or IL-1β, they can differentiate into immunogenic mature DC (mDC). By their part, mDC are capable of priming and activating T cells to initiate an immune response after providing the three required activation signals of the immune synapsis once a specific and immunogenic antigen has been recognized. During this maturation process, an upregulation of the expression of human leukocyte antigen (HLA) molecules, as well as, of other costimulatory molecules such as CD40, CD80, CD83, or CD86 takes place, along with an increase in the production of IL-12 and other proinflammatory cytokines (2, 3, 8).

However, a third type of DC has been defined in the last years, combining immune tolerance-inducing properties with a stability against maturation stimuli, called tolerogenic DC (tolDC). It is not clear whether tolDC constitute a different DC subset by themselves or if they are mere maturation-impaired iDC, although there seems to be a consensus about which features they have to possess in order to develop their regulatory function. Thus, tolDC usually present one or more of these characteristics: a semi-mature phenotype, with low expression of co-stimulatory (CD80, CD86, CD83) and HLA molecules, a maintained CCR7-dependant migratory ability toward the secondary lymphoid organs, an increased IL-10 production accompanied by low or null IL-12 and IFN-γ secretion, a lowered T cell-proliferation priming capability, potential to induce Treg and stability against maturation in front of a proinflammatory milieu. Specifically the latter, which has been described in the majority of these studies, probably constitutes the most important feature among all of them (16–18).

Importantly, tolDC can be differentiated *in vitro* from peripheral blood monocytes in the presence of a determined tolerogenic-inducing agent. Indeed, a wide variety of protocols have emerged in the last 20 years describing the induction of tolDC with several stimuli, such as anti-inflammatory cytokines—IL-10 (19, 20), TGF-β (20, 21)—, pharmacological agents and immunosuppressant compounds—rapamycin (20, 22, 23), different corticosteroids (24), dexamethasone (20, 23, 25, 26), vitamin D3 (20, 23, 27) or a combination of both dexamethasone and vitamin D3 (28)—, several drugs and blocking molecules—aspirin (29), mitomycin C (30), the NF-κB inhibitor BAY11-7082 (11)—and other strategies, such as genetic engineering for the selective repression or induction of key molecules and pathways (10, 31), among many others further discussed below. Generally, most of these protocols share several features in common, such as the differentiation of monocytes in the presence of GM-CSF and IL-4, as well as, the addition of a maturation stimulus (which usually includes different combinations of LPS, monophosphoryl lipid A, TNF-α, IL-1β, prostaglandin E2, and/or IL-6), with few exceptions.

Either if we assume tolDC are a specific DC subset *per se* or just a modified state of iDC, there must be some footprint left by this condition. At the transcriptomic level, as already hinted, some obvious downregulated candidates would be the genes encoding co-stimulatory molecules or pro-inflammatory cytokines. However, those features would be shared with steady state iDC, thus making them useless in terms of differentially characterizing tolDC. In fact, ideally, a comparison against both immature and immunogenic control conditions should be taken into account in the search of specific genetic biomarkers, something that has not been considered in the majority of the reviewed studies. An ideal candidate should be, furthermore, clearly differentiated by a matter of full induction or repression, as a slight increase/decrease of its expression could be ambiguous and would always require the use of robust controls, which is not always possible.

Consequently, many research groups have been working on the identification of genetic markers for human tolDC, and deep transcriptomic studies are becoming more frequent each year. However, and although several studies have described a pool of markers for some specific tolerogenic cell products, common genetic biomarkers have not been found yet.

### Glucocorticoids and immunomodulatory molecules in the generation of tolerogenic dendritic cells

Since mDC are immunogenic cells, or, in other words, promoters of inflammatory responses, the use of corticosteroids and other immunosuppressant drugs has been widely reported for the generation of tolDC. Rapamycin (20, 22, 23, 32, 33) and a combination of hydrocortisone and clobetasol-17-propionate (24), but especially dexamethasone (20, 23, 25, 26, 32–45), have all been used for the generation of tolDC. As a glucocorticoid-induced molecule, the expression of the gene encoding the anti-inflammatory mediator known as glucocorticoid-induced leucine zipper (GILZ) (46) has been reported strongly up-modulated in many of these studies, thus making it a good albeit predictable marker for tolDC generated with this kind of immunomodulatory agents. Furthermore, other molecules related with the complement and the immune system have been found commonly up- or down-modulated in several of these tolDC protocols, such as the anti-inflammatory cytokine IL-10 (up-regulated), the pro-inflammatory cytokine IL-12 or the fascin 1-encoding gene *FSCN1* (both down-modulated), which are common features that define these cells (32). The full list of differentially expressed molecules reported for each of the abovementioned protocols and their respective references can be found on Table 1.

**Table 1:** Differentially up- and down-modulated genes and proteins in the most reported human tolDC-inducing protocols.

	**Protocol**	**Type**	**Up-modulated molecules**	**Down-modulated molecules**	**References**
tolDC	Dexamethasone	Gene	***ANXA1, C1QA, C1QC, C1QTNF1, C3AR1, CCL17, CD163, CD300LF, CD32, CFH, CLIC2, CSGALNACT1, CTSC, DCR3, EP2, EP3, F13A, FCGR2A, FCGR2B, FKBP5, FOXO3, FPR1, GILZ, GPX1, IDO1, IL10**, IL12A, IL27B, **IMDH2, JAG1, MERTK, MRC1, MT1, NCF1, OSF1, P2RY14, SLC39A8, SOD2, STAB1, TPP1, ZBTB16***	***CCL22, CD1C, FCER1A, IDO1**, IL12B, **LAMP3, MMP12, ZNF366***	(26, 32, 34–36, 38, 39, 41)
		Protein	**CYP1B1, DAB2, DPYD, FCER1G, FCGR3A, FTL, GCLC, IVNS1ABP, LRRC25, MCTP1**, MERTK**, NUDT16, PDCD4, PECAM1, RNASE6, RNASET2, SIGLEC5, SLCO2B1**	**FSCN1**	(12, 34, 37)
		miRNA	*miR-328-5P, miR-638, miR-663, miR-762, miR-1275, miR-1228, miR-1909*	*miR-142-5p*	(40)
	Dexamethasone + rosiglitazone	Gene	***FABP4, GILZ***		(47)
	Dexamethasone + vitamin D2	Protein	**ERK1/2, IDO, JNK/SAPK, mTOR, p38 MAPK, STAT3**		(48)
	Dexamethasone + vitamin D3	Gene	*ACADM, ACADVL, ACO1, ACO2, ACOX2, ACSS1, ALDH2, ATP5G3, ATP5J, ATP5O, BLVRB, C1orf162, C1QA, CCR5, **CD14**, CD209, CD274, **CD52**, CLIC1, COX11, COX6A1, COX7A2, CTSB, CTSD, CTSH, CYC1, DHRS9, EIF3B, EIF3C, EIF3CL, EIF4A3, FBP1, FCGR2B, FCGR3A, FN1, FTH1, FTL, G6PD, GAPDH, IDH3A, IDH3B, **ILT3**, LDHB, LILRB4, MATK, MCEMP1, MDH2, ME1, ME3, NDUFB9, NDUFS1, NDUFS8, NOS3, PCK2, PDHA1, PDXK, PIK3R1, PKM2, PNP, PRDX3, PTPN6, RAC2, RGCC, RPS12, RPS19, RPS21, RPS6KA1, RPS6KA2, SDHA, SLC11A1, SLC27A5, SLC2A1, SLC2A5, SNCA, SUCLG1, SUCLG2, TCEB1, **TGFB1**, TP53, TPI1, UQCR10, UQCR11, UQCRB, UQCRC1*	*ACTB, ADAM12, ADAM19, ANKRD33B, AOC1, **CD25**, **CD40**, **CD80**, **CD83**, **CD86**, DPYSL2, EHF, **FSCN1**, GPR157, ICOSLG, IKZF1, IKZF4, IL12B, IL2RA, ORMDL3, PIK3CG, PLEKHA5, PPP1R16B, PTPN2, SH2B3, TYK2, WDR1*	(49–51)
		Protein	ADK, AKR1A1, ALDH2, ALDOA, ATP5H, ECHS1, FBP1, FTL, G6PD, GPD2, GALK, MPDH2, PGAM, PGM1, PKM2, PNP, PRDX6, TALDO1, TKT, TPI1	DPYSL2, ENO1, FSCN1, HSPD1, PDIA3	(37)
	Hepatocyte growth factor	Gene	***IL10***		(52)
	IFN-γ	Gene		***IRF4, RELB, IL12p40***	(53, 54)
	IL-10	Gene	***ANXA1, C1QC, CTSB, CTSC, CTSL, F13A, FTH1, GILZ, HLA-DOB, IL8, LILRB3, MRC1, STAB1, THBS1, TPP1***	***CD74, LAMP3***	(32, 41, 55)
	IL-10 + IL-6	Gene	***CTSB, CTSL, FTH1, HLA-DOB, IL-8, THBS1***	***CD74***	(55)
	Poly I:C	Gene	***IDO1, PDL1***		(56, 57)
	Rapamycin	Gene	***ANXA1, C1QC, CTSC, GILZ, GPX1, IMDH2, OSF1, TPP1***	***RALDH1***	(32)
	Retinoic acid	Gene	***ALDH1A1, ALDH1A2, CD141, GARP***		(58, 59)
	TGF-β	Gene	***ANXA1, CTSL, CXCL1, CXCR3, FTH1, HLA-DOB, IL8, LILRB3, THBS1***	***CD74, STAB1***	(32, 55)
	TX527 (vitamin D3 analog)	Protein	ACADVL, ACO2, ACOX1, ATP5A1, CTSD, CTSS, COPG, FBP1, G6PD, HADHA, IDH3A, MnSOD, OGDH, PCK2, PKM2, PRX3, PTM, UQCRFS1	ACAT1, ARCN1, DLD, PA28beta, PTM, RabGDI	(60)
	Vitamin D3	Gene	***ALOX5, ATP5A1, CAMP, CCL22, CD14, CD300LF, CMYC, CYP24, CYP24A1, CYP27B1, GILZ, GLUT3, HK3, ILT3, IRF8, LDHA, LGALS9, PDHA1, PFKFB4, PIK3CG, PRKAA1, THBD, VDR***	***CD1A, CD1C, CD1E, CD36, CD80, F13A, IER3, IRF4, LAMP3***	(32, 36, 41, 61–63)
		Protein	**AKT, FTL, GSK-3b, mTOR**	**FSCN1, SOD2**	(37)
		miRNA	*miR-378*		(64)

*Genes validated by qPCR or proteins validated by western blot are shown in bold*.

Dexamethasone-induced tolDC (dexa-tolDC) are one of the most widely implemented approaches worldwide for the generation of human tolDC, and are being or have been tested on clinical trials for the treatment of numerous autoimmune diseases, such as Crohn's disease (http://www.clinicaltrials.gov, NCT02622763) (12), rheumatoid arthritis (http://www.clinicaltrials.gov, NCT03337165; NCT03337165) and both multiple sclerosis or neuromyelitis optica (http://www.clinicaltrials.gov, NCT02283671). Several studies have reported the differential up-modulation of genes *C1QA* (encoding the C1q complement protein, chain A) (34, 35), *CD163* (34, 35), *GILZ* (32, 35, 36), *MERTK* (encoding the MER Proto-Oncogene Tyrosine Kinase, also used as a marker in the abovementioned clinical trial for Crohn's disease) (12, 26, 35) and *ZBTB16* (encoding zinc finger and BTB domain containing protein 16) (34, 35) in dexa-tolDC, thus making them the most relevant candidate biomarkers for this specific protocol. Additionally, the differential expression of *IDO1*, the gene encoding the indoleamine 2,3-dioxygenase —a molecule widely related to the induction of immune tolerance (65)—, has also been reported in dexa-tolDC. However, there is some controversy in this regard, as it has been found both up- (35) and down-modulated (32) in different studies. Besides, other induced genes described in studies using dexamethasone, relevant by their role in the modulation and mediation of different mechanisms of the immune system—with their respective encoded proteins in brackets—, are *CD300LF* (CD300 molecule-like, family member F), *F13A* (coagulation factor XIII A), *FCGR2B* (Fc fragment of IgG receptor IIb), *FCGR3A* (Fc fragment of IgG receptor IIIa), *MRC1* (mannose receptor C-type 1), and *STAB1* (stabilin 1), as well as, other non-immune related genes like *FTL* (ferritin light chain), *IMDH2* (inosine monophosphate dehydrogenase 2), and *SOD2* (superoxide dismutase 2). Furthermore, the combination of dexamethasone with rosiglitazone has also been reported for the generation of tolDC, highlighting the induction of *FABP4* (fatty acid-binding protein 4) with this protocol, but specially also of *GILZ* gene (47).

The generation of human rapamycin-modulated tolDC (rapa-tolDC) is the second most reported protocol of this group of pharmacological and immunomodulatory agents. However, transcriptomic studies in tolDC generated with this strong immunosuppressant drug are not as predominant as those induced with dexamethasone. Yet, several genes have been postulated as candidate biomarkers for rapa-tolDC, both immune-related—*ANXA1* (annexin 1), *C1QC, CTSC* (cathepsin C) and *GILZ*—and non-immune-related —*GPX1* (Glutathione Peroxidase 1), *IMDH2, OSF1* (pleiotrophin) and *TPP1* (tripeptidyl peptidase 1)—. Interestingly, all these genes have also been described in common with dexa-tolDC (32).

Additionally, the immunostimulant TLR3 ligand polyinosinic:polycytidylic acid (poly I:C) has also been reported to induce human tolDC, although in an inconsistent and poorly efficient manner. Nevertheless, the differential up-modulation of both *IDO1* and *PD-L1*, two genes involved in the induction and maintenance of immune tolerance, has been confirmed by quantitative PCR for these cells (56, 57). As for tolDC induced with hydrocortisone and clobetasol-17-propionate, no transcriptomic biomarkers have been reported.

### Vitamins A and D modulate the transcriptomic footprint of tolerogenic dendritic cells

As reviewed by Mora et al. (66), vitamins A and D exert important immunomodulatory properties. While vitamin A and specifically its metabolite, retinoic acid, have been reported to have an influence in T cell differentiation and proliferation, as well as, Treg induction, vitamin D plays an important role as an immunoregulatory agent in the inhibition of T cell proliferation and the reduction of IL-2 and IFN-γ secretion. Furthermore, the absence or low levels of vitamin D in the organism has been widely linked to an increase in the incidence of autoimmune diseases.

The tolerogenic-inducing properties of 1,25-dihydroxycholecalciferol, the active form of vitamin D3, over DC (vitD3-tolDC) have been widely reported *in vitro* in many studies performed with murine (67–70) and even cattle cells (71), although we will only focus on biomarkers of human vitD3-tolDC (20, 23, 27, 32, 33, 36, 37, 41, 44, 61–64, 72–74). As a measurement of its relevance, such is the importance of vitD3-tolDC in the field of tolerogenic cell products that even two clinical trials are already ongoing for the treatment of multiple sclerosis using this cell product in Badalona, Spain (http://www.clinicaltrials.gov, NCT02903537) and in Antwerp, Belgium (http://www.clinicaltrials.gov, NCT02618902). Several transcriptomic and proteomic pre-clinical studies in human vitD3-tolDC have evidenced several genes and proteins strongly induced with this approach, including immune-related molecules—*CCL22* (62, 63), *ILT3* (immunoglobulin-like transcript 3) (36), *CD300LF* (62) or *GILZ* (32), these last two in common with dexa-tolDC—and oxidative metabolism enzymes and regulators—*GLUT3* (glucose transporter 3), *LDHA* (lactate dehydrogenase A), *mTOR* (mammalian target of rapamycin), *PDHA1* (pyruvate dehydrogenase E1, subunit alpha 1) or *PFKFB4* (fructose-2,6-bisphosphatase) (63)—, as well as direct targets of the response to vitamin D3 through the interaction with its receptor, like *CYP24A1* (cytochrome P450, family 24, subfamily A, member 1) (41, 61–63) and of course *VDR* (vitamin D receptor) (41). By their part, the repression of several co-stimulatory, pro-inflammatory, and antigen presenting genes and molecules like *CD1A, CD1C, CD80, FSCN1* or the transcription factor *IRF4* has been reported at the transcriptomic and proteomic levels (37, 62). Additionally, a synthetic structural analog of vitamin D3, TX527, has also been used for the induction of human tolDC (60). However, and although the up-modulation of the ATP synthase F1 subunit alpha-encoding gene (*ATP5A1*) was reported in common with vitD3-tolDC, the transcriptomic resemblance was more relevant with tolDC induced with a combination of dexamethasone and vitamin D3, a strategy that will be further discussed in the next section. Nevertheless, some of these induced molecules consist of mostly metabolic-related genes—*ACADVL* (Acyl-CoA dehydrogenase very long chain), *ACO2* (aconitase 2), *FBP1* (fructose bisphosphatase 1), *IDH3A* (isocitrate dehydrogenase 3, subunit alpha), *PCK2* (phosphoenolpyruvate carboxykinase 2) and *PKM2* (pyruvate kinase M2)—and *CTSD*, encoding the protease cathepsin D (37, 49, 50).

The use of vitamin A-derived molecules like retinoic acid, however, has not been so widely reported for the generation of human tolDC and only the selective up-regulation of *ALDH1A1* and *ALDH1A2* genes, encoding the aldehyde dehydrogenase 1 family members A1 and A2—involved the metabolism of retinoic acid—has been reported, as well as, the induction of *CD141* and *GARP* genes (58, 59). Other differentially expressed genes induced by the protocols mentioned in this section are shown in Table 1.

### The synergic effect of dexamethasone and vitamin D

Since dexamethasone and vitamin D treatments alone are able to generate tolDC, the combination of both of them is expected to induce synergic effects that would strengthen the tolerogenic functionality of these cells. Consequently, the simultaneous use of dexamethasone and vitamin D3, or vitamin D2 in a few cases (48, 75), has become one of the most widely reported human tolDC-generating protocols *in vitro*. Indeed, these cells have even reached the clinical phase for the treatment of rheumatoid arthritis, with successful results regarding the safety and tolerability of the product (http://www.clinicaltrials.gov, NCT01352858) (13).

**Figure 1:**
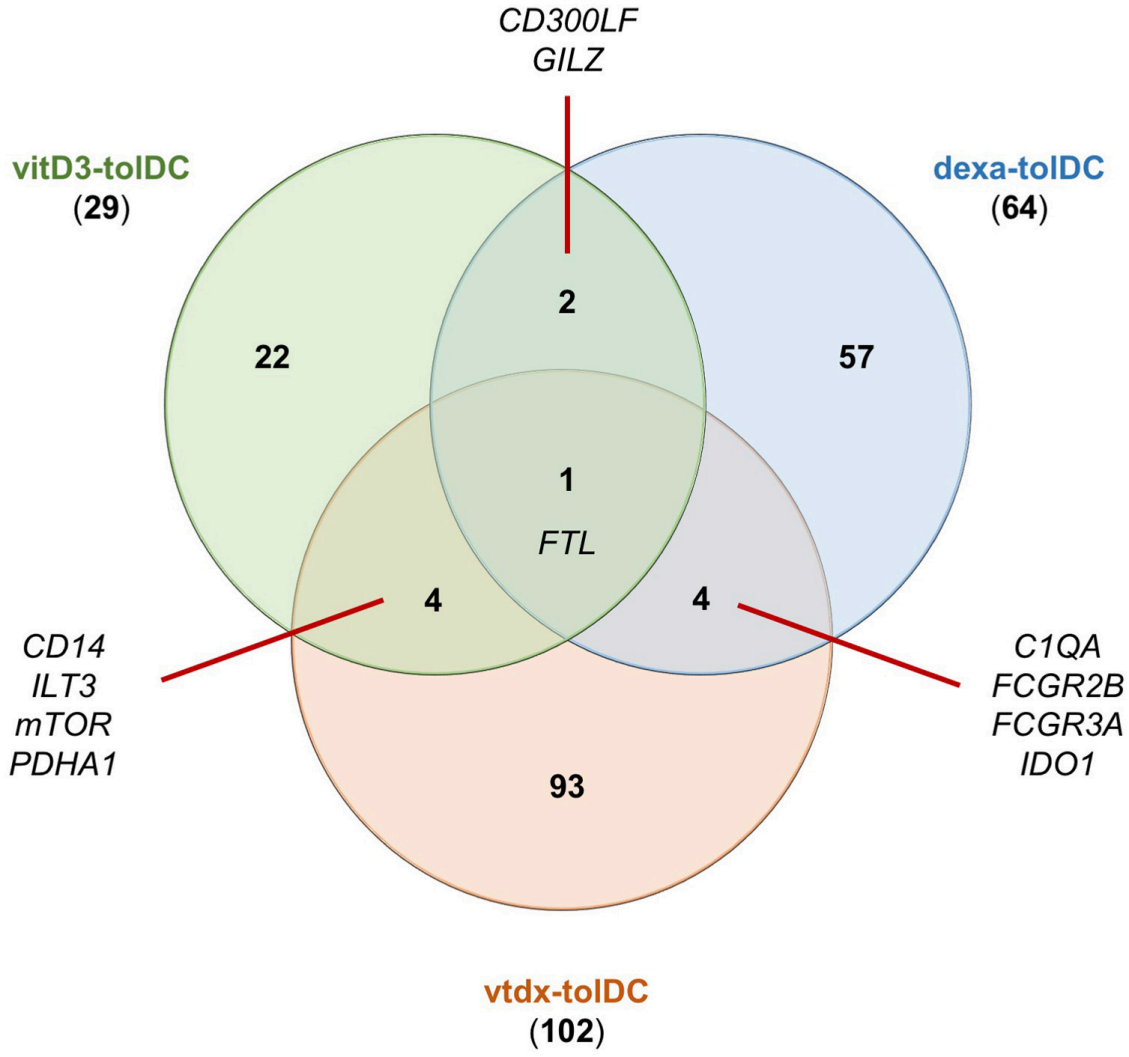
Common up-modulated genes in tolDC induced with either vitamin D3, dexamethasone or the combination of both. The numbers in the Venn diagram indicate the number of reportedly induced genes for each condition alone or in combination with one or both of the others. Dexa-tolDC: dexamethasone-induced tolDC; vitD3-tolDC: vitamin D3-induced tolDC; vtdx-tolDC: dexamethasone + vitamin D3-induced tolDC.

As expected, the genetic signature of dexamethasone + vitamin D-induced tolDC (vtdx-tolDC) reported in pre-clinical studies partially overlaps with that reported for each or both of these treatments alone to generate human dexa- and vitD3-tolDC. In fact, the analysis of the reported data for these protocols showed that *C1QA, FCGR2B, FCGR3A* and *IDO1* genes were found induced in common with dexa-tolDC (34, 35, 38, 50, 48) and *CD14, ILT3, mTOR* and *PDHA1* were shared with vitD3-tolDC (36, 48–50, 62, 63). Nevertheless, our analysis evidenced that the up-regulation of *FTL* and the suppression of *FCSN1* genes were the only genetic modulations in common between these three protocols (34, 37, 63, 50). Interestingly, the function of the proteins encoded by all these genes is strongly related to the modulation of the immune system. Surprisingly, however, there was a pool of genes that were only described for vtdx-tolDC but not for either dexa-tolDC nor vitD3-tolDC, such as *CTSB, DHRS9* (dehydrogenase/reductase 9), *FTH1* (ferritin heavy chain 1), *RGCC* (regulator of cell cycle), *SLC11A1* (solute carrier family 11 member 1), *TBET* or *TGFB1* (49, 50, 51). Indeed, after our study, it is worth noting that out of 64 up-modulated genes and/or proteins reported for dexa-tolDC, 29 genes for vitD3-tolDC and 102 genes for vtdx-tolDC, only 4 genes could be found in common between vtdx-tolDC and each treatment separately, as shown in the Venn diagram in Figure 1. The chances are, however, that many of these genes could simply not be detected or were overlooked in the validation process of the separated protocols due to intrinsic limitations of the methodologies used, as it is known that biases frequently appear in high throughput transcriptomic and proteomic techniques. For this same reason, for instance, some already mentioned immune-related and metabolic genes were detected simultaneously induced in vtdx-tolDC and tolDC generated in the presence of the vitamin D3 analog TX527—*ACADVL, ACO2, CTSD, FBP1, G6PD* (glucose-6-phosphate dehydrogenase), *IDH3A, PCK2, PKM2*)—(37, 60, 49, 50). Although the down-modulation of genes is not as relevant toward the identification of transcriptomic biomarkers, it is nonetheless worth noting that the *FSCN1* gene has been found repressed in vtdx-tolDC, dexa-tolDC, and vitD3-tolDC at the same time (37, 50). Table 1 shows a complete list of the differentially expressed genes and proteins reported in protocols using a combination of dexamethasone and vitamin D derivates.

### The effect of cytokines and growth factors in the induction of tolerogenic dendritic cells

Many different kinds of cytokines have been used for the induction of human tolDC, ranging from anti-inflammatory—IL-10 (19, 20, 32, 33, 41, 55, 76–78), TGF-β (21, 32, 33, 55, 79) or both (80)—to even immunostimulatory molecules—IFN-γ (53, 54) or a combination of IL-6 with IL-10 (55)—, but also several growth factors—hepatocyte growth factor (52) and low-doses of GM-CSF alone (81).

As previously mentioned, the secretion of IL-10 is one of the most sought features of tolDC due to its anti-inflammatory and regulatory properties. Consequently, the generation of tolDC in the presence of exogenous IL-10 (IL10-tolDC) constitutes one of the most implemented protocols for the generation of this type of regulatory cell products. In fact, many of the genes and molecules already cited for other protocols, with immune or metabolic involvement, have also been found induced in IL10-tolDC, such as, *ANXA1, C1QC, CTSB, CTSC, CTSL* (cathepsin L), *F13A, FTH1, HLA-DOB, IL-8, LILRB3* (leukocyte immunoglobulin-like receptor B3), *MRC1, STAB1, THBS1, TPP1* and, especially for its repeated prevalence, *GILZ* (32, 55). Also, and in line with the traditional concept of tolDC, the down-modulation of the antigen presenting molecule *CD74* (also known as HLA-DR) (55) and *LAMP3* (lysosomal-associated membrane protein 3), typically found on iDC (41), has been reported. Interestingly, the combined exposure in front of both of IL-10 and IL-6 for the generation of tolDC performed in one of the previously cited articles did not seem to change the transcriptomic profile of these cells, as many of the above mentioned genes were also found accordingly induced or repressed like they were in IL10-tolDC (55).

The use of TGF-β for the *in vitro* differentiation of tolDC is not as widely established as IL-10, but still some potential biomarkers have been described, both exclusively for this product (the immune related-genes *CXCL1* and *CXCR3*) and in common with other regulatory cells (*ANXA1, CTSL, FTH1, HLA-DOB, IL-8, LILRB3, THBS1*) (32, 55). Just like in IL10-tolDC, *CD74* appears differentially repressed in TGF-β-induced tolDC but, controversially, also does *STAB1*, reportedly up-modulated in the former protocol (55). As far as we are concerned, no potential transcriptomic or proteomic markers have been reported in cells induced with the combination of IL-10 and TGF-β for the generation of human tolDC.

Surprisingly, IFN-γ has also been described in a couple of publications for the generation of tolDC, even though it does not constitute the most obvious strategy due to its proinflammatory properties. Nevertheless, these studies have reported the selective reduction in the expression of the pro-inflammatory genes *IRF4, RELB*, and *IL12p40* in this cell product (53, 54). Consequently, the down-modulation of these genes is in line with the expected anti-inflammatory profile for tolDC, and even *IRF4* has also been reported as differentially repressed in vitD3-tolDC, as mentioned above (62). All the biomarkers described within the protocols mentioned in this section are shown in Table 1.

Finally, the differentiation of stable tolDC from monocytes in the presence of low doses of GM-CSF, and in the absence of IL-4 in the culture, has also been reported in humans (81), but also in animal models (82–84). In fact, their clinical use is being tested under the context of a multicentre trial named *The ONE Study ATDC* in living-donor renal transplantation (http://www.clinicaltrials.gov, NCT02252055) (85). However, any potential biomarker in human low-dose GM-CSF-induced tolDC has been reported yet.

### Genetic changes induced in tolerogenic dendritic cells generated with other strategies

The pharmacological agents and factors mentioned so far comprise the most predominant strategies in the literature for the induction of tolDC, but there is still a wide variety of drugs, proteins and several treatments with the potential of generating this type of regulatory DC products. However, provided that the aim of this review is to look for universal biomarkers of immune tolerance, we have also considered these approaches. In fact, a significant amount of studies have reported the differential expression of several genes and molecules that could become potential biomarkers for their respective and specific protocols, generating tolDC in the presence of different organic compounds—such as the *Aspergillus* cell wall (32), curcumin (86), mitomycin C (87), paeoniflorin (88), phosphatidylserine liposomes imitating apoptotic bodies (89)—, other cell types—mast cells (90) and trophoblasts (91)—and a variety of agents, conditions and/or molecules—for instance a combination of the complement protein C5a and LPS (92), seminal plasma (93), the Wnt-5a protein (94) or even the deprivation of tryptophan in the culture (95)—. However, there are still many other different strategies without transcriptomic or proteomic studies reported in the literature that are, therefore, outside of the objective of this review. The full list of differentially expressed genes and molecules in the protocols mentioned in this section is presented in Table 2.

**Table 2:** Differentially up- and down-modulated genes and proteins in other human tolDC- and Mreg-inducing protocols.

	**Protocol**	**Type**	**Up-modulated molecules**	**Down-modulated molecules**	**References**
tolDC	*Aspergillus* cell wall	Gene	***ANXA1, STAB1, GILZ, IDO, RALDH1, RALDH2***	***F13A, MRC1***	(32)
	C5a and LPS	Gene	***RGCC, FERMT2, SLC39A14, TNFSF14, TGFB1***	***IL12B, FOXO1***	(92)
	Curcumin	Gene		***RELB***	(86)
	Mast cells	Gene	***IDO1, NFKB1, NFKB2, RELB, SOCS5***	***SOCS3***	(90)
	Mitomycin C	Gene	***ADM, CSF2RA, DDIT3, FDXR, GAB2, LILRB4, LRDD, MAFB, MAP4K4, PERP, TNFRSF10B, TRAF4, TSC22D3***	***CFLAR*** **(FLAME-1, I-FLICE, Usurpin)**, ***NRG2***	(87)
	Paeoniflorin	Gene	**IDO1**		(88)
	Phosphatidyl-serine lyposomes	Gene	*CLCN6, **CYTH4**, IFNLR1, LAIR1, LDLR, MFSD2A, NFKBIA, PLAUR, PPME1, SHB, SLC43A3, TNFAIP3, **TNFSF14**, VEGFA*	*ALKBH1, ATP10D, AURKA, BCL2L1, BLCAP, BST1, BTBD3, BTK, BUB1, C9orf64, CASP3, CBX4, CD1D, CDC23, CDC42SE1, CDK13, CDYL2, CKAP2, CLCN3, CSRP2BP, CUL3, DAPP1, DCAF12, DCAF7, DCLRE1A, DCTD, DDO, DYRK2, EHBP1, ERLIN1, FBXO25, FBXO36, FRAT2, FZD5, GIMAP4, GLRX, GOLPH3L, GTF2B, HHEX, HPGD, ICK, KBTBD6, KIF11, KIF20B, LMNB1, LNX2, MAPRE2, MCM4, MCPH1, MDM1, MEF2C, MEGF9, MIER3, MLH1, MNDA, MSH2, MYB, N4BP1, NCAPG2, NET1, NFIA, NSMCE4A, NUP160, PAQR8, PARG, PAXIP1, PCNA, PMP22, PROS1, RAB32, RAD51C, RCSD1, RMDN1, RMND5A, SCYL3, SEC22C, SKI, SLAMF6, SLC10A7, SLC40A1, SMC2, SNN, SNX18, SOCS2, STIM2, STX3, TIMMDC1, TNFRSF11A, TPK1, TRIM5, UBE2E3, UBFD1, UNC50, VWA5A, WRNIP1, ZBED3, ZBTB39, ZBTB5, ZFP36L2, ZNF436*	(89)
	Seminal plasma	Gene	***COX2, TGFB1***	***CD1A***	(93)
	Trophoblasts	Gene	***IDO1***		(91)
	Tryptophan-deprived	Gene	***CHOP, ILT3***		(95)
	Wnt5a	Gene	***ID3, IRF1, IRF2, SOCS3, TLR1***	***ID2, IRF8, TLR3, TLR4, TLR5***	(94)
Mreg	M-CSF + IFN-γ	Gene	***ALDH1A1, ALDH1A2, CD1D, DHRS9***		(96)
	M-CSF + LPS	Protein		**IL-12p35, IL-12p40, IL23-p19**	(97)

*Genes validated by qPCR or proteins validated by western blot are shown in bold*.

A totally different approach to generate tolDC consists in using targeted genetic engineering in order to achieve cells with specific functional features either silenced or induced. There are several strategies reported in this regard, ranging from the impairment of immunogenic properties—such as silencing the expression of *CD40, CD80*, and *CD86*, already tested in type 1 diabetes patients, which was the first clinical trial using a tolerogenic cell therapy (10, 98) (http://www.clinicaltrials.gov, NCT00445913)—to selectively inducing the production of several anti-inflammatory cytokines like IL-10 and TGF-β (31, 99), overexpressing the IL-12 and IL-23-suppressor factor *SOCS-3* (100) or transfecting the cells with a modified *CTLA4* construct that inhibits the expression of the co-stimulatory molecules CD80 and CD86 (101). Surprisingly, some approaches using genetic manipulation achieved to generate human IL-10-producing DC through the induction of, *a priori*, immunogenic functions such as the CD40-CD40L signaling pathway (102). However, the definition of transcriptomic biomarkers for tolDC induced by genetic engineering would not be of much utility, provided that the differentially expressed genes or proteins to check would be precisely those that have been specifically induced or repressed by the procedure itself.

## Generation of regulatory macrophages: differences and similarities with tolerogenic dendritic cells

Mreg constitute one of the three main macrophage subtypes, being the other two the classical macrophages and the so-called alternatively activated macrophages, or M2 macrophages. However, and as reviewed by Fleming and Mosser (103), Mreg present unique features: they are characterized by their ability to modulate the immune system toward a regulatory T_H_2 response through the production of IL-10 and a limited or absent secretion of IL-12 mediated by the activation of the ERK cascade. In addition, these cells present an increased antigen-presenting functionality with an elevated expression of HLA class II and B7 co-stimulatory molecules. And this is, probably, the crucial feature in which Mreg and M2 macrophages differ the most, because although both subsets exhibit immunoregulatory properties, the ability to induce antigen-specific responses is limited in M2 macrophages due to their low HLA expression. However, Mreg are considered to deploy their potent T cell suppressor functions mainly through three non-antigen-specific mechanisms: via IFN-γ-induced IDO activity, by a contact-dependent deletion of activated T lymphocytes or mediating the induction of TIGIT^+^ FoxP3^+^ Treg (9, 96, 104, 105).

Just like tolDC, human macrophages can be generated *in vitro* by differentiating them from monocytes. Briefly, classical macrophages are obtained in the presence of GM-CSF, and M2 and Mreg macrophages are generated in the presence of M-CSF, but with different supplementary treatments. While M2 macrophages are normally achieved using M-CSF + IL-4 and/or other T_H_2 cytokines, Mreg are treated with M-CSF + LPS or IFN-γ for a brief period of time (106, 107). This combination of M-CSF and a short and complementary pro-inflammatory treatment is precisely the responsible for the strong induction of IL-10 production, something that both stimuli alone fail to achieve in macrophages (108, 109).

The generation of human Mreg is not as widely extended as tolDC, and consequently the number of protocols describing the differentiation of these cells is much more reduced. However, several molecules have already been postulated as potential biomarkers for these cell products. So far, transcriptomic studies have only been performed over LPS (LPS-Mreg) and IFN-γ-activated Mreg (IFN-Mreg). The former, LPS-Mreg, were initially described as IL-10-producing M2 macrophages, and their impaired IL-12 and IL-23 production was confirmed by qPCR (97, 110). However, IFN-Mreg are more widely reported and studied, especially considering their translation into the clinic, where they have already been used for the treatment of living-donor renal transplant-recipient patients (http://www.clinicaltrials.gov, NCT00223067 and NCT02085629) (9, 105). This product is obtained by the stimulation of M-CSF-differentiated macrophages with IFN-γ, after 7 days of culture (9, 105, 111), and a strong up-modulation of *ALDH1A1, ALDH1A2* and *CD1D* genes has been reported, as well as the induction of *DHRS9* as an specific IFN-Mreg biomarker (96). The detailed list of markers reported in pre-clinical human Mreg protocols is shown in Table 2.

Nevertheless, although the list of genetic biomarkers described in Mreg is short, the identification of *DHRS9* in IFN-Mreg achieves a high relevance in the context of immune tolerance biomarkers, provided that the enzyme encoded by these gene seems to be involved in the biosynthesis of retinoic acid (112). As commented above, this compound is a vitamin A-derived molecule that can be used to differentiate human monocytes into tolDC. Interestingly, both *ALDH1A1* and *ALDH1A2* genes have been identified as differentially induced in retinoic acid-generated tolDC (58) as well as IFN-Mreg, making them two interesting candidates for the characterization of at least this couple of different tolerance-inducing cell products. Furthermore, the differential up-modulation of *DHRS9* has also been reported in vtdx-tolDC, also discussed above (49). Consequently, since these cells are generated with both dexamethasone and vitamin D3, a clear relation between the transcriptomic profile of both IFN-Mreg and tolDC induced with either vitamin A or D is likely to exist. For this reason, further studies and validations in this direction could be of great interest, as potential common biomarkers of two different immune-regulatory myeloid cell-derived products could be identified.

## Summary and concluding remarks

The identification of robust biomarkers for the characterization of tolerogenic and immunoregulatory cell products constitutes one of the last steps needed to take the final leap toward the broad application of these novel autologous antigen-specific therapies in the clinic. Specifically, their key importance resides in their capability to provide a fast and reliable quality control of the proper generation, functionality and safety of tolDC and Mreg.

In this article we have performed an exhaustive review of the currently published human tolDC- and Mreg-generating protocols that have reported potential biomarkers for these cells, with the aim of elucidating if a common transcriptomic or proteomic pattern relating all of them could be drawn. However, as it has been discussed, albeit many genes and molecules have been found separately induced using different strategies to generate these immunoregulatory cell products, so far, there is not a biomarker or a pool of biomarkers that can functionally characterize or at least identify the entirety of the studied protocols. Nonetheless, this is not necessarily bad news, as the chances of identifying a common biomarker were slim given the overwhelming variety of approaches and cell types reported in this review. As already mentioned above, the immune system can deploy several strategies for the induction of tolerance that modulate many different immune and non-immune related pathways and transcriptomic cascades, thus making this goal even more unlikely. However, it is also worth noting that finding biomarkers provided only by the tolerance-inducing mechanisms could also be misleading; for instance, DC subtypes like iDC are capable of developing some tolerogenic functions, but still they could not be applied as a therapeutic approach in autoimmune diseases provided their lack of stability against pro-inflammatory stimuli, as discussed above.

Still, despite the consideration of such a wide variety and heterogenicity of protocols for the induction of regulatory cells, a significant amount of differentially expressed genes encoding several anti-inflammatory and immunomodulatory molecules has been reported in very different protocols, for instance *IDO1* (in 7 approaches) *GILZ* (in 6 approaches) or *ANXA1* (in 5 approaches). Similarly, the down-modulation of the pro-inflammatory cytokine IL-12 has been reported in 5 different tolDC-inducing strategies. In other words, in this review we have gathered all the genes and proteins that have been described separately with each of the approaches for the generation of tolDC and Mreg in the literature, and we have subsequently compared and put them all together in order to evidence potential common biomarkers between them. The complete list of the genes that have been reported in studies with at least two different approaches for the generation of human tolDC and/or Mreg are shown in Table 3. Therefore, the general idea that lies behind these reported molecules is that all the considered tolerogenic-inducing agents are modulating the cells toward a regulatory profile that might be partially shared between some approaches, but that is often achieved through different mechanisms and biological pathways that are strongly dependent on the stimuli used to generate them.

**Table 3:** Differentially expressed genes reported in at least two different protocols for the generation of human tolDC and/or Mreg.

**Gene**	**Name**	**Modulation**	**Repeats**	**Protocols**	**References**
*ACADVL*	Acyl-CoA Dehydrogenase Very Long Chain	Up	2	Dexa+vitD3, TX527	(60, 49)
*ACO2*	Aconitase 2	Up	2	Dexa+vitD3, TX527	(60, 49)
*ALDH1A1*	Retinaldehyde Dehydrogenase 1	Up	3	Asp, IFNg Mreg, RA	(58, 96)
*ALDH1A2*	Retinaldehyde Dehydrogenase 2	Up	2	IFNg Mreg, RA	(58, 96)
*ANXA1*	Annexin A1	Up	5	Asp, dexa, IL10, rapa, TGFb	(32)
*ATP5A1*	ATP Synthase 5 Alpha Subunit 1	Up	2	TX527, vitD3	(63, 60)
*C1QA*	Complement C1q A Chain	Up	2	Dexa, dexa+vitD3	(34, 35, 50)
*C1QC*	Complement C1q C Chain	Up	3	Dexa, IL10, rapa	(32)
*CD14*	Cluster of Differentiation 14	Up	2	dexa+vitD3, vitD3	(62, 50)
*CD1A*	CD1a Receptor	Down	2	Sem, vitD3	(50, 93)
*CD1C*	CD1c Receptor	Down	2	Dexa, vitD3	(35, 62)
*CD300LF*	CD300 Molecule Like Family Member F	Up	2	Dexa, vitD3	(34, 62)
*CD80*	Cluster of Differentiation 80	Down	2	Dexa+vitD3, vitD3	(62, 50)
*CTSB*	Cathepsin B	Up	3	Dexa+vitD3, IL10, IL10+6	(50, 55)
*CTSC*	Cathepsin C	Up	3	Dexa, IL10, rapa	(32)
*CTSD*	Cathepsin D	Up	2	Dexa+vitD3, TX527	(60, 49)
*CTSL*	Cathepsin L	Up	3	IL10, IL10+6, TGFb	(55)
*DHRS9*	Dehydrogenase/Reductase 9	Up	2	Dexa+vitD3, IFNg Mreg	(49, 96)
*F13A*	Coagulation Factor XIII A	Up	2	Dexa, IL10	(32)
		Down	2	Asp, vitD3	(32)
*FBP1*	Fructose-Bisphosphatase 1	Up	2	Dexa+vitD3, TX527	(37, 60, 49)
*FCGR2B*	Fc Fragment Of IgG Receptor IIb	Up	2	Dexa, dexa+vitD3	(34, 49)
*FCGR3A*	Fc Fragment Of IgG Receptor IIIa	Up	2	Dexa, dexa+vitD3	(34, 49)
*FSCN1*	Fascin Actin-Bundling Protein 1	Down	3	Dexa, dexa+vitD3, vitD3	(37, 50)
*FTH1*	Ferritin Heavy Chain	Up	4	Dexa+vitD3, IL10, IL10+6, TGFb	(50, 55)
*FTL*	Ferritin Light Chain	Up	3	Dexa, dexa+vitD3, vitD3	(34, 37, 50)
*G6PD*	Glucose-6-Phosphate Dehydrogenase	Up	2	Dexa+vitD3, TX527	(37, 60, 50)
*GILZ*	Glucocorticoid-Induced Leucine Zipper	Up	6	Asp, dexa, RGZ, IL10, rapa, vitD3	(32, 35, 36, 47)
*GPX1*	Glutathione Peroxidase 1	Up	2	Dexa, rapa	(32)
*HLA-DOB*	Human Leukocyte Antigen Class II, DO Beta Chain	Up	3	IL10, IL10+6, TGFb	(55)
*IDH3A*	Isocitrate Dehydrogenase 3 Alpha	Up	2	Dexa+vitD3, TX527	(60, 49)
*IDO1*	Indoleamine 2,3-Dioxygenase	Up	7	Asp, dexa, dexa+vitD2, mast, pae, pIC, tropho	(32, 35, 57, 48, 88, 90, 91)
*IL-10*	Interleukin 10	Up	2	Dexa, hepa	(35, 52)
*IL-12*	Interleukin 12	Down	5	C5a, dexa, dexa+vitD3, IFNg, LPS Mreg	(35, 38, 50, 53, 54, 92, 97)
*IL-8*	Interleukin 8	Up	2	IL10, IL10+6	(55)
*ILT3*	Immunoglobulin-Like Transcript 3	Up	4	Dexa+vitD3, mitC, tryp, vitD3	(36, 50, 87, 95)
*IMDH2*	Inosine Monophosphate Dehydrogenase 2	Up	2	Dexa, rapa	(32)
*IRF4*	Interferon Regulatory Factor 4	Down	2	IFNg, vitD3	(62, 54)
*LAMP3*	Lysosome-Associated Membrane Protein 3	Down	3	Dexa, IL10, vitD3	(41)
*LILRB3*	Leukocyte Immunoglobulin Like Receptor B3	Up	2	IL10, TGFb	(55)
*MRC1*	Mannose Receptor C-Type 1	Up	2	Dexa, IL10	(32)
*mTOR*	Mammalian Target Of Rapamycin	Up	2	Dexa+vitD2, vitD3	(63, 48)
*OSF1*	Pleiotrophin	Up	2	Dexa, rapa	(32)
*PCK2*	Phosphoenolpyruvate Carboxykinase 2	Up	2	Dexa+vitD3, TX527	(60, 49)
*PDHA1*	Pyruvate Dehydrogenase E1 Alpha 1 Subunit	Up	2	Dexa+vitD3, vitD3	(60, 49)
*PIK3CG*	Phosphatidylinositol-3-Kinase Subunit Gamma	Up	2	Dexa+vitD3, vitD3	(63, 49, 50)
*PKM2*	Pyruvate Kinase Muscle Isozyme M2	Up	2	Dexa+vitD3, TX527	(37, 60, 49)
*RELB*	RelB Transcription Factor, NF-κB Subunit	Down	2	Cur, IFNg	(53, 54, 86)
*RGCC*	Regulator Of Cell Cycle	Up	2	C5a, dexa+vitD3	(50, 92)
*STAB1*	Stabilin 1	Up	3	Asp, dexa, IL10	(32)
*TGFB*	Transforming Growth Factor Beta	Up	3	C5a, dexa+vitD3, sem	(51, 92, 93)
*THBS1*	Thrombospondin 1	Up	3	IL10, IL10+6, TGFb	(55)
*TNFSF14*	TNF Superfamily Member 14	Up	2	C5a, lipo	(88, 92)
*TPP1*	Tripeptidyl Peptidase 1	Up	3	Dexa, IL10, rapa	(32)

*The column “Modulation” indicates if a determined gene has been found up- or down-modulated, and the field “Repeats” indicates the amount of different protocols in which each gene or protein has been described. The abbreviations stand for either tolDC induced with asp, Aspergillus cell wall; C5a, C5a, and LPS; cur, curcumin; dexa, dexamethasone; dexa+vitD2, dexamethasone + vitamin D2; dexa+vitD3, dexamethasone + vitamin D3; hepa, hepatocyte growth factor; IFNg, IFN-γ; IL10, IL-10; IL10-6, IL-6 + IL-10; mast, mast cells; mitC, mitomycin C; pae, paeoniflorin; pIC, Polyinosinic:polycytidylic acid; RA, retinoic acid; rapa, rapamycin; RGZ, rosiglitazone; sem, seminal plasma; TGFb, TGF-β; tropho, trophoblasts; tryp, tryptophan deprivation; vitD3, vitamin D3; or regulatory macrophages induced with IFNg Mreg, IFN-γ; LPS Mreg, lipopolysaccharide*.

Consequently, this review evidences that the definition of strong biomarkers for tolDC and Mreg is still needed, but also that, although a universal transcriptomic profile of immune tolerance induction might not be achievable, the elaboration of useful panels of biomarkers can still be feasible for determined pools of tolerogenic products. Bearing that in mind, our work could therefore serve as a starting point for developing and guiding further research in this field. For instance, one of the next steps that could be taken in this regard could be to specifically try to validate some of the above discussed genes in different protocols in which they have not been explicitly reported, either because they have been already identified in several approaches—like *IDO1* or *GILZ*—or because the stimuli used to induce the tolerogenic status share some functional or structural resemblance that might translate into the induction of common pathways and metabolic processes. In other words, with this review we intend to provide a useful reference of currently described biomarkers from which direct the investigation of new genes and proteins, most likely protocol-specific.

Thus, the combination of both stimulus-specific and some other partially-common differentially expressed genes could potentially lead to the development of transcriptomic panels of tolerogenic functionality. After all, provided that the relevance of tolerance-inducing cell therapies in the treatment of autoimmune diseases and solid organ transplantation rejection is becoming hugely relevant in the last years, the need for adequate and objective biomarkers is increasing accordingly. And in this context, the definition of panels of tolerogenic functionality for at least a limited pool of protocols would consequently provide a robust tool for the establishment of reliable quality and safety controls for trials using tolDC- and/or Mreg-based therapies in the near future, which would also allow to properly compare them and therefore to dramatically accelerate their translation into the clinic.

## Author contributions

EM-C, JN-B, and MM conceived the manuscript. JN-B wrote the manuscript. EM-C and MM reviewed the manuscript.

### Conflict of interest statement

The authors declare that the research was conducted in the absence of any commercial or financial relationships that could be construed as a potential conflict of interest.

## References

[1] Matzinger P. The danger model: a renewed sense of self. Science (2002) 296:301–5. 10.1126/science.1071059 11951032

[2] Banchereau J Steinman RM. Dendritic cells and the control of immunity. Nature (1998) 392:245–52. 10.1038/32588 9521319

[3] Banchereau J Briere F Caux C Davoust J Lebecque S Liu Y-J. Immunobiology of Dendritic Cells. Annu Rev Immunol. (2000) 18:767–811. 10.1146/annurev.immunol.18.1.767 10837075

[4] Mueller DL. Mechanisms maintaining peripheral tolerance. Nat Immunol. (2010) 11:21–7. 10.1038/ni.1817 20016506

[5] Ganguly D Haak S Sisirak V Reizis B. The role of dendritic cells in autoimmunity. Nat Rev Immunol. (2013) 13:566–77. 10.1038/nri3477 23827956 PMC4160805

[6] Hopp A-K Rupp A Lukacs-Kornek V. Self-antigen presentation by dendritic cells in autoimmunity. Front Immunol. (2014) 5:55. 10.3389/fimmu.2014.00055 24592266 PMC3923158

[7] Ovchinnikov Dmitry A. Macrophages in the embryo and beyond: much more than just giant phagocytes. Genesis (2008) 46:447–62. 10.1002/dvg.20417 18781633

[8] Hilkens CMU Isaacs JD Thomson AW. Development of dendritic cell-based immunotherapy for autoimmunity. Int Rev Immunol. (2010) 29:156–183. 10.3109/08830180903281193 20199240

[9] Hutchinson JA Riquelme P Sawitzki B Tomiuk S Miqueu P Zuhayra M. Cutting edge: immunological consequences and trafficking of human regulatory macrophages administered to renal transplant recipients. J Immunol. (2011) 187:2072–8. 10.4049/jimmunol.1100762 21804023

[10] Giannoukakis N Phillips B Finegold D Harnaha J Trucco M. Phase I (safety) study of autologous tolerogenic dendritic cells in type 1 diabetic patients. Diabetes Care (2011) 34:2026–32. 10.2337/dc11-0472 21680720 PMC3161299

[11] Benham H Nel HJ Law SC Mehdi AM Street S Ramnoruth N. Citrullinated peptide dendritic cell immunotherapy in HLA risk genotype-positive rheumatoid arthritis patients. Sci Transl Med. (2015) 7:290ra87. 10.1126/scitranslmed.aaa9301 26041704

[12] Jauregui-Amezaga A Cabezón R Ramírez-Morros A España C Rimola J Bru C. Intraperitoneal administration of autologous tolerogenic dendritic cells for refractory crohn's disease: a phase I study. J Crohns Colitis (2015) 9:1071–8. 10.1093/ecco-jcc/jjv144 26303633

[13] Bell GM Anderson AE Diboll J Reece R Eltherington O Harry RA. Autologous tolerogenic dendritic cells for rheumatoid and inflammatory arthritis. Ann Rheum Dis. (2017) 76:227–34. 10.1136/annrheumdis-2015-208456 27117700 PMC5264217

[14] Ten Brinke A Hilkens CMU Cools N Geissler EK Hutchinson JA Lombardi G Clinical use of tolerogenic dendritic cells-harmonization approach in european collaborative effort. Med Inflamm. (2015) 2015:471719 10.1155/2015/471719 PMC4706930 26819498

[15] Satpathy AT Wu X Albring JC Murphy KM. Re(de)fining the dendritic cell lineage. Nat Immunol (2012) 13:1145–54. 10.1038/ni.2467 23160217 PMC3644874

[16] Adler HS Steinbrink K Tolerogenic dendritic cells in health and disease: friend and foe! Eur J Dermatol EJD (2007) 17:476–91. 10.1684/ejd.2007.0262 17951127

[17] Morelli AE Thomson AW. Tolerogenic dendritic cells and the quest for transplant tolerance. Nat Rev Immunol. (2007) 7:610–21. 10.1038/nri2132 17627284

[18] Suwandi JS Nikolic T Roep BO. Translating mechanism of regulatory action of tolerogenic dendritic cells to monitoring endpoints in clinical trials. Front Immunol. (2017) 8:1598. 10.3389/fimmu.2017.01598 29250062 PMC5715363

[19] Steinbrink K Wölfl M Jonuleit H Knop J Enk AH. Induction of tolerance by IL-10-treated dendritic cells. J Immunol. (1997) 159:4772–80. 9366401

[20] Boks MA Kager-Groenland JR Haasjes MSP Zwaginga JJ van Ham SM Ten Brinke A. IL-10-generated tolerogenic dendritic cells are optimal for functional regulatory T cell induction–a comparative study of human clinical-applicable DC. Clin Immunol. (2012) 142:332–42. 10.1016/j.clim.2011.11.011 22225835

[21] Fogel-Petrovic M Long JA Misso NL Foster PS Bhoola KD Thompson PJ. Physiological concentrations of transforming growth factor β1 selectively inhibit human dendritic cell function. Int Immunopharmacol. (2007) 7:1924–33. 10.1016/j.intimp.2007.07.003 18039529

[22] Fedoric B Krishnan R. Rapamycin downregulates the inhibitory receptors ILT2, ILT3, ILT4 on human dendritic cells and yet induces T cell hyporesponsiveness independent of FoxP3 induction. Immunol Lett. (2008) 120:49–56. 10.1016/j.imlet.2008.06.009 18652845

[23] Naranjo-Gómez M Raïch-Regué D Oñate C Grau-López L Ramo-Tello C Pujol-Borrell R. Comparative study of clinical grade human tolerogenic dendritic cells. J Transl Med. (2011) 9:89. 10.1186/1479-5876-9-89 21658226 PMC3141500

[24] de Jong EC Vieira PL Kalinski P Kapsenberg ML Corticosteroids inhibit the production of inflammatory mediators in immature monocyte-derived DC and induce the development of tolerogenic DC3. J Leukoc Biol. (1999) 66:201–4. 10449154 10.1002/jlb.66.2.201

[25] Xia C-Q Peng R Beato F Clare-Salzler MJ. Dexamethasone induces IL-10-producing monocyte-derived dendritic cells with durable immaturity. Scand J Immunol. (2005) 62:45–54. 10.1111/j.1365-3083.2005.01640.x 16091124

[26] Cabezón R Carrera-Silva EA Flórez-Grau G Errasti AE Calderón-Gómez E Lozano JJ España C. MERTK as negative regulator of human T cell activation. J Leukoc Biol. (2015) 97:751–60. 10.1189/jlb.3A0714-334R 25624460 PMC4370049

[27] Penna G Adorini L. 1 Alpha,25-dihydroxyvitamin D3 inhibits differentiation, maturation, activation, and survival of dendritic cells leading to impaired alloreactive T cell activation. J Immunol. (2000) 164:2405–11. 10.4049/jimmunol.164.5.2405 10679076

[28] Anderson AE Sayers BL Haniffa MA Swan DJ Diboll J Wang X-N. Differential regulation of naïve and memory CD4+ T cells by alternatively activated dendritic cells. J Leukoc Biol. (2008) 84:124–33. 10.1189/jlb.1107744 18430785 PMC2504714

[29] Buckland M Jago C Fazekesova H George A Lechler R Lombardi G. Aspirin modified dendritic cells are potent inducers of allo-specific regulatory T-cells. Int Immunopharmacol. (2006) 6:1895–1901. 10.1016/j.intimp.2006.07.008 17219690

[30] Jiga LP Bauer TM Chuang J-J Opelz G Terness P. Generation of tolerogenic dendritic cells by treatment with mitomycin C: inhibition of allogeneic T-cell response is mediated by downregulation of ICAM-1, CD80, and CD86. Transplantation (2004) 77:1761–4. 10.1097/01.TP.0000131165.37177.6E 15201679

[31] Lu L Lee WC Takayama T Qian S Gambotto A Robbins PD. Genetic engineering of dendritic cells to express immunosuppressive molecules (viral IL-10, TGF-beta, and CTLA4Ig). J Leukoc Biol. (1999) 66:293–6. 10449170 10.1002/jlb.66.2.293

[32] Zimmer A Bouley J Le Mignon M Pliquet E Horiot S Turfkruyer M. A regulatory dendritic cell signature correlates with the clinical efficacy of allergen-specific sublingual immunotherapy. J Allergy Clin Immunol. (2012) 129:1020–30. 10.1016/j.jaci.2012.02.014 22464673

[33] Adnan E Matsumoto T Ishizaki J Onishi S Suemori K Yasukawa M. Human tolerogenic dendritic cells generated with protein kinase C inhibitor are optimal for functional regulatory T cell induction — a comparative study. Clin Immunol. (2016) 173:96–108. 10.1016/j.clim.2016.09.007 27658741

[34] Gueguen C Bouley J Moussu H Luce S Duchateau M Chamot-Rooke J. Changes in markers associated with dendritic cells driving the differentiation of either TH2 cells or regulatory T cells correlate with clinical benefit during allergen immunotherapy. J Allergy Clin Immunol. (2016) 137:545–58. 10.1016/j.jaci.2015.09.015 26522402

[35] García-González PA Schinnerling K Sepúlveda-Gutiérrez A Maggi J Mehdi AM Nel HJ. Dexamethasone and monophosphoryl lipid a induce a distinctive profile on monocyte-derived dendritic cells through transcriptional modulation of genes associated with essential processes of the immune response. Front Immunol. (2017) 8:1350. 10.3389/fimmu.2017.01350 29109727 PMC5660598

[36] Chamorro S García-Vallejo JJ Unger WWJ Fernandes RJ Bruijns SCM Laban S. TLR triggering on tolerogenic dendritic cells results in TLR2 up-regulation and a reduced proinflammatory immune program. J Immunol. (2009) 183:2984–94. 10.4049/jimmunol.0801155 19648269

[37] Ferreira GB Kleijwegt FS Waelkens E Lage K Nikolic T Hansen DA. Differential protein pathways in 1,25-dihydroxyvitamin d(3) and dexamethasone modulated tolerogenic human dendritic cells. J Proteome Res. (2012) 11:941–71. 10.1021/pr200724e 22103328

[38] Dixon KO van der Kooij SW Vignali DAA van Kooten C. Human tolerogenic dendritic cells produce IL-35 in the absence of other IL-12 family members. Eur J Immunol. (2015) 45:1736–47. 10.1002/eji.201445217 25820702 PMC4617619

[39] Flórez-Grau G Cabezón R Borgman KJE España C Lozano JJ Garcia-Parajo MF Up-regulation of EP2 and EP3 receptors in human tolerogenic dendritic cells boost the immunosuppressive activity of PGE2. J Leukoc Biol. (2017) 102:881–95. 10.1189/jlb.2A1216-526R 28630103

[40] Lombardi V Luce S Moussu H Morizur L Gueguen C Neukirch C. Effector and regulatory dendritic cells display distinct patterns of miRNA expression. Immun Inflamm Dis. (2017) 5:310–7. 10.1002/iid3.165 28497578 PMC5569363

[41] Malaguarnera L Marsullo A Zorena K Musumeci G Di Rosa M. Vitamin D3 regulates LAMP3 expression in monocyte derived dendritic cells. Cell Immunol. (2017) 311:13–21. 10.1016/j.cellimm.2016.09.013 27697285

[42] García-González P Morales R Hoyos L Maggi J Campos J Pesce B. A short protocol using dexamethasone and monophosphoryl lipid A generates tolerogenic dendritic cells that display a potent migratory capacity to lymphoid chemokines. J Transl Med. (2013) 11:128. 10.1186/1479-5876-11-128 23706017 PMC3674980

[43] Escobar A Aguirre A Guzmán MA González R Catalán D Acuña-Castillo C. Tolerogenic dendritic cells derived from donors with natural rubber latex allergy modulate allergen-specific T-cell responses and IgE production. PLoS ONE (2014) 9:e85930. 10.1371/journal.pone.0085930 24465795 PMC3899084

[44] Spallanzani RG Torres NI Avila DE Ziblat A Iraolagoitia XLR Rossi LE. Regulatory dendritic cells restrain NK cell IFN-γ production through mechanisms involving NKp46, IL-10, and MHC class I–specific inhibitory receptors. J Immunol. (2015) 195:2141–8. 10.4049/jimmunol.1403161 26232426

[45] Maggi J Schinnerling K Pesce B Hilkens CM Catalán D Aguillón JC. Dexamethasone and monophosphoryl lipid A-modulated dendritic cells promote antigen-specific tolerogenic properties on naive and memory CD4+ T Cells. Front Immunol. (2016) 7:359. 10.3389/fimmu.2016.00359 27698654 PMC5027201

[46] Ronchetti S Migliorati G Riccardi C. GILZ as a mediator of the anti-inflammatory effects of glucocorticoids. Front Endocrinol. (2015) 6:170. 10.3389/fendo.2015.00170 26617572 PMC4637413

[47] Obreque J Vega F Torres A Cuitino L Mackern-Oberti JP Viviani P. Autologous tolerogenic dendritic cells derived from monocytes of systemic lupus erythematosus patients and healthy donors show a stable and immunosuppressive phenotype. Immunology (2017) 152:648–59. 10.1111/imm.12806 28763099 PMC5680052

[48] Dánová K Klapetková A Kayserová J Šedivá A Špíšek R Jelínková LP. NF-κB, p38 MAPK, ERK1/2, mTOR, STAT3 and increased glycolysis regulate stability of paricalcitol/dexamethasone-generated tolerogenic dendritic cells in the inflammatory environment. Oncotarget (2015) 6:14123–38. 10.18632/oncotarget.4234 26053099 PMC4546455

[49] Malinarich F Duan K Hamid RA Bijin A Lin WX Poidinger M. High mitochondrial respiration and glycolytic capacity represent a metabolic phenotype of human tolerogenic dendritic cells. J Immunol. (2015) 194:5174–86. 10.4049/jimmunol.1303316 25917094

[50] Nikolic T Woittiez NJC van der Slik A Laban S Joosten A Gysemans C. Differential transcriptome of tolerogenic versus inflammatory dendritic cells points to modulated T1D genetic risk and enriched immune regulation. Genes Immun. (2017) 18:176–83. 10.1038/gene.2017.18 28794505

[51] Anderson AE Swan DJ Wong OY Buck M Eltherington O Harry RA. Tolerogenic dendritic cells generated with dexamethasone and vitamin D3 regulate rheumatoid arthritis CD4^+^T cells partly via transforming growth factor-β1. Clin Exp Immunol. (2017) 187:113–23. 10.1111/cei.12870 27667787 PMC5167049

[52] Rutella S Bonanno G Procoli A Mariotti A de Ritis DG Curti A. Hepatocyte growth factor favors monocyte differentiation into regulatory interleukin (IL)-10++IL-12low/neg accessory cells with dendritic-cell features. Blood (2006) 108:218–27. 10.1182/blood-2005-08-3141 16527888

[53] Rojas D Krishnan R. IFN-γ generates maturation-arrested dendritic cells that induce T cell hyporesponsiveness independent of Foxp3^+^ T-regulatory cell generation. Immunol Lett. (2010) 132:31–7. 10.1016/j.imlet.2010.05.003 20580745

[54] Rojas-Canales D Krishnan R Jessup CF Coates PT. Early exposure of interferon-γ inhibits signal transducer and activator of transcription-6 signalling and nuclear factor κB activation in a short-term monocyte-derived dendritic cell culture promoting ‘FAST' regulatory dendritic cells. Clin Exp Immunol. (2012) 167:447–58. 10.1111/j.1365-2249.2011.04537.x 22288588 PMC3374277

[55] Torres-Aguilar H Aguilar-Ruiz SR González-Pérez G Munguía R Bajaña S Meraz-Ríos MA. Tolerogenic dendritic cells generated with different immunosuppressive cytokines induce antigen-specific anergy and regulatory properties in memory CD4^+^ T cells. J Immunol. (2010) 184:1765–75. 10.4049/jimmunol.0902133 20083662

[56] Gröschel S Piggott KD Vaglio A Ma-Krupa W Singh K Goronzy J. TLR-mediated induction of negative regulatory ligands on dendritic cells. J Mol Med. (2008) 86:443–55. 10.1007/s00109-008-0310-x 18253710 PMC2556182

[57] Pavlović B Tomić S Dokić J Vasilijić S Vučević D Lukić J. Fast dendritic cells matured with Poly (I:C) may acquire tolerogenic properties. Cytotherapy (2015) 17:1763–76. 10.1016/j.jcyt.2015.08.001 26455276

[58] Bakdash G Vogelpoel LT van Capel TM Kapsenberg ML de Jong EC. Retinoic acid primes human dendritic cells to induce gut-homing, IL-10-producing regulatory T cells. Mucosal Immunol. (2015) 8:265–78. 10.1038/mi.2014.64 25027601

[59] Agrawal S Ganguly S Tran A Sundaram P Agrawal A. Retinoic acid treated human dendritic cells induce T regulatory cells via the expression of CD141 and GARP which is impaired with age. Aging (2016) 8:1223–35. 10.18632/aging.100973 27244900 PMC4931828

[60] Ferreira GB van Etten E Lage K Hansen DA Moreau Y Workman CT. Proteome analysis demonstrates profound alterations in human dendritic cell nature by TX527, an analogue of vitamin D. Proteomics (2009) 9:3752–64. 10.1002/pmic.200800848 19639594

[61] Penna G Amuchastegui S Giarratana N Daniel KC Vulcano M Sozzani S 1,25-Dihydroxyvitamin D3 selectively modulates tolerogenic properties in myeloid but not plasmacytoid dendritic cells. J Immunol. (2007) 178:145–53. 10.4049/jimmunol.178.1.145 17182549

[62] Széles L Keresztes G Töröcsik D Balajthy Z Krenács L Póliska S. 1,25-dihydroxyvitamin D3 is an autonomous regulator of the transcriptional changes leading to a tolerogenic dendritic cell phenotype. J Immunol. (2009) 182:2074–83. 10.4049/jimmunol.0803345 19201860

[63] Ferreira GB Vanherwegen A-S Eelen G Gutiérrez ACF Van Lommel L Marchal K Vitamin D3 induces tolerance in human dendritic cells by activation of intracellular metabolic pathways. Cell Rep. (2015) 10:711–25. 10.1016/j.celrep.2015.01.013 25660022

[64] Pedersen AW Holmstrøm K Jensen SS Fuchs D Rasmussen S Kvistborg P Phenotypic and functional markers for 1α,25-dihydroxyvitamin D3-modified regulatory dendritic cells. Clin Exp Immunol. (2009) 157:48–59. 10.1111/j.1365-2249.2009.03961.x 19659770 PMC2710592

[65] Mbongue JC Nicholas DA Torrez TW Kim N-S Firek AF Langridge WHR. The role of indoleamine 2, 3-dioxygenase in immune suppression and autoimmunity. Vaccines (2015) 3:703–29. 10.3390/vaccines3030703 26378585 PMC4586474

[66] Mora JR Iwata M von Andrian UH. Vitamin effects on the immune system: vitamins A and D take centre stage. Nat Rev Immunol. (2008) 8:685–98. 10.1038/nri2378 19172691 PMC2906676

[67] Ferreira GB Gysemans CA Demengeot J da Cunha JP Vanherwegen AS Overbergh L. 1,25-dihydroxyvitamin D3 promotes tolerogenic dendritic cells with functional migratory properties in NOD mice. J Immunol. (2014) 192:4210–20. 10.4049/jimmunol.1302350 24663679

[68] Huang Y Zhao Y Ran X Wang C Increased expression of herpesvirus entry mediator in 1,25-dihydroxyvitamin D3-treated mouse bone marrow-derived dendritic cells promotes the generation of CD4^+^CD25^+^Foxp3^+^ regulatory T cells. Mol Med Rep. (2014) 9:813–8. 10.3892/mmr.2013.1874 24366217

[69] Mansilla MJ Sellès-Moreno C Fàbregas-Puig S Amoedo J Navarro-Barriuso J Teniente-Serra A. Beneficial effect of tolerogenic dendritic cells pulsed with MOG autoantigen in experimental autoimmune encephalomyelitis. CNS Neurosci Ther. (2015) 21:222–30. 10.1111/cns.12342 25403984 PMC6495243

[70] Mansilla MJ Contreras-Cardone R Navarro-Barriuso J Cools N Berneman Z Ramo-Tello C. Cryopreserved vitamin D3-tolerogenic dendritic cells pulsed with autoantigens as a potential therapy for multiple sclerosis patients. J Neuroinflamm. (2016) 13:113. 10.1186/s12974-016-0584-9 27207486 PMC4874005

[71] Corripio-Miyar Y Mellanby RJ Morrison K McNeilly TN. 1,25-Dihydroxyvitamin D3 modulates the phenotype and function of Monocyte derived dendritic cells in cattle. BMC Vet Res. (2017) 13:390. 10.1186/s12917-017-1309-8 29237505 PMC5729451

[72] Raïch-Regué D Naranjo-Gómez M Grau-López L Ramo C Pujol-Borrell R Martínez-Cáceres E. Differential effects of monophosphoryl lipid A and cytokine cocktail as maturation stimuli of immunogenic and tolerogenic dendritic cells for immunotherapy. Vaccine (2012) 30:378–87. 10.1016/j.vaccine.2011.10.081 22085546

[73] Raïch-Regué D Grau-López L Naranjo-Gómez M Ramo-Tello C Pujol-Borrell R Martínez-Cáceres E Stable antigen-specific T-cell hyporesponsiveness induced by tolerogenic dendritic cells from multiple sclerosis patients. Eur J Immunol. (2012) 42:771–82. 10.1002/eji.201141835 22488365

[74] Lee W-P Willekens B Cras P Goossens H Martínez-Cáceres E Berneman ZN. Immunomodulatory effects of 1,25-dihydroxyvitamin D3 on dendritic cells promote induction of T cell hyporesponsiveness to myelin-derived antigens. J Immunol Res. (2016) 2016:5392623. 10.1155/2016/5392623 27703987 PMC5039280

[75] Dánová K Grohová A Strnadová P Funda DP Šumník Z Lebl J. Tolerogenic dendritic cells from poorly compensated type 1 diabetes patients have decreased ability to induce stable antigen-specific T cell hyporesponsiveness and generation of suppressive regulatory T cells. J Immunol. (2017) 198:729–40. 10.4049/jimmunol.1600676 27927966

[76] Bellinghausen I König B Böttcher I Knop J Saloga J. Inhibition of human allergic T-helper type 2 immune responses by induced regulatory T cells requires the combination of interleukin-10-treated dendritic cells and transforming growth factor-beta for their induction. Clin Exp Allergy J Br Soc Allergy Clin Immunol. (2006) 36:1546–55. 10.1111/j.1365-2222.2006.02601.x 17177678

[77] Bellinghausen I Reuter S Martin H Maxeiner J Luxemburger U Türeci Ö. Enhanced production of CCL18 by tolerogenic dendritic cells is associated with inhibition of allergic airway reactivity. J Allergy Clin Immunol. (2012) 130:1384–93. 10.1016/j.jaci.2012.08.039 23102918

[78] Kryczanowsky F Raker V Graulich E Domogalla MP Steinbrink K. IL-10–modulated human dendritic cells for clinical use: identification of a stable and migratory subset with improved tolerogenic activity. J Immunol. (2016) 197:3607–17. 10.4049/jimmunol.1501769 27683749

[79] Abediankenari S Ghasemi M. Generation of immune inhibitory dendritic cells and CD4+T regulatory cells inducing by TGF-beta. Iran J Allergy Asthma Immunol. (2009) 8:25–30. 10.08.01/ijaai.2530 19279356

[80] Torres-Aguilar H Blank M Kivity S Misgav M Luboshitz J Pierangeli SS. Tolerogenic dendritic cells inhibit antiphospholipid syndrome derived effector/memory CD4^+^ T cell response to β2GPI. Ann Rheum Dis. (2012) 71:120–8. 10.1136/annrheumdis-2011-200063 21914629

[81] Chitta S Santambrogio L Stern LJ. GMCSF in the absence of other cytokines sustains human dendritic cell precursors with T cell regulatory activity and capacity to differentiate into functional dendritic cells. Immunol Lett. (2008) 116:41–54. 10.1016/j.imlet.2007.11.013 18166231

[82] Segovia M Cuturi MC Hill M. Preparation of mouse bone marrow-derived dendritic cells with immunoregulatory properties. Methods Mol Biol. (2011) 677:161–8. 10.1007/978-1-60761-869-0_11 20941609

[83] Baas MC Kuhn C Valette F Mangez C Duarte MS Hill M. Combining autologous dendritic cell therapy with CD3 antibodies promotes regulatory T cells and permanent islet allograft acceptance. J Immunol. (2014) 193:4696–703. 10.4049/jimmunol.1401423 25252962

[84] Pletinckx K Vaeth M Schneider T Beyersdorf N Hünig T Berberich-Siebelt F. Immature dendritic cells convert anergic nonregulatory T cells into Foxp3- IL-10^+^ regulatory T cells by engaging CD28 and CTLA-4. Eur J Immunol. (2015) 45:480–91. 10.1002/eji.201444991 25382658

[85] Geissler EK. The ONE study compares cell therapy products in organ transplantation: introduction to a review series on suppressive monocyte-derived cells. Transplant Res. (2012) 1:11. 10.1186/2047-1440-1-11 23369457 PMC3561076

[86] Rogers NM Kireta S Coates PTH. Curcumin induces maturation-arrested dendritic cells that expand regulatory T cells *in vitro* and *in vivo*. Clin Exp Immunol. (2010) 162:460–73. 10.1111/j.1365-2249.2010.04232.x 21070208 PMC3026549

[87] Terness P Oelert T Ehser S Chuang JJ Lahdou I Kleist C. Mitomycin C-treated dendritic cells inactivate autoreactive T cells: toward the development of a tolerogenic vaccine in autoimmune diseases. Proc Natl Acad Sci USA. (2008) 105:18442–7. 10.1073/pnas.0807185105 19017789 PMC2584573

[88] Chen D Li Y Wang X Li K Jing Y He J. Generation of regulatory dendritic cells after treatment with paeoniflorin. Immunol Res. (2016) 64:988–1000. 10.1007/s12026-015-8773-7 26721806

[89] Rodriguez-Fernandez S Pujol-Autonell I Brianso F Perna-Barrull D Cano-Sarabia M Garcia-Jimeno S. Phosphatidylserine-liposomes promote tolerogenic features on dendritic cells in human type 1 diabetes by apoptotic mimicry. Front Immunol. (2018) 9:253. 10.3389/fimmu.2018.00253 29491866 PMC5817077

[90] Rodrigues CP Ferreira ACF Pinho MP de Moraes CJ Bergami-Santos PC Barbuto JAM. Tolerogenic IDO+ dendritic cells are induced by PD-1-expressing mast cells. Front Immunol. (2016) 7:9. 10.3389/fimmu.2016.00009 26834749 PMC4724729

[91] Salamone G Fraccaroli L Gori S Grasso E Paparini D Geffner J. Trophoblast cells induce a tolerogenic profile in dendritic cells. Hum Reprod. (2012) 27:2598–606. 10.1093/humrep/des208 22718280

[92] Zaal A Nota B Moore KS Dieker M van Ham SM Ten Brinke A. TLR4 and C5aR crosstalk in dendritic cells induces a core regulatory network of RSK2, PI3Kβ, SGK1, and FOXO transcription factors. J Leukoc Biol. (2017) 102:1035–54. 10.1189/jlb.2MA0217-058R 28733463

[93] Lenicov FR Rodrigues CR Sabatté J Cabrini M Jancic C Ostrowski M Semen promotes the differentiation of tolerogenic dendritic cells. J Immunol. (2012) 189:4777–86. 10.4049/jimmunol.1202089 23066152

[94] Valencia J Hernández-López C Martínez VG Hidalgo L Zapata AG Vicente Á. Wnt5a skews dendritic cell differentiation to an unconventional phenotype with tolerogenic features. J Immunol. (2011) 187:4129–39. 10.4049/jimmunol.1101243 21918189

[95] Brenk M Scheler M Koch S Neumann J Takikawa O Häcker G. Tryptophan deprivation induces inhibitory receptors ILT3 and ILT4 on dendritic cells favoring the induction of human CD4^+^CD25^+^ Foxp3^+^ T regulatory cells. J Immunol. (2009) 183:145–54. 10.4049/jimmunol.0803277 19535644

[96] Riquelme P Amodio G Macedo C Moreau A Obermajer N Brochhausen C. DHRS9 is a stable marker of human regulatory macrophages. Transplantation (2017) 101:2731–8. 10.1097/TP.0000000000001814 28594751 PMC6319563

[97] Verreck FA de Boer T Langenberg DM Hoeve MA Kramer M Vaisberg E. Human IL-23-producing type 1 macrophages promote but IL-10-producing type 2 macrophages subvert immunity to (myco)bacteria. Proc Natl Acad Sci USA. (2004) 101:4560–5. 10.1073/pnas.0400983101 15070757 PMC384786

[98] Di Caro V Phillips B Engman C Harnaha J Trucco M Giannoukakis N Retinoic acid-producing, *ex-vivo*-generated human tolerogenic dendritic cells induce the proliferation of immunosuppressive B lymphocytes. Clin Exp Immunol. (2013) 174:302–17. 10.1111/cei.12177 23865694 PMC3828834

[99] Gorczynski RM Bransom J Cattral M Huang X Lei J Xiaorong L. Synergy in induction of increased renal allograft survival after portal vein infusion of dendritic cells transduced to express TGFβ and IL-10, along with administration of CHO Cells expressing the regulatory molecule OX-2. Clin Immunol. (2000) 95:182–9. 10.1006/clim.2000.4860 10866124

[100] Li Y Chu N Rostami A Zhang G-X. Dendritic cells transduced with SOCS-3 exhibit a tolerogenic/DC2 phenotype that directs type 2 Th cell differentiation *in vitro* and *in vivo*. J Immunol. (2006) 177:1679–88. 10.4049/jimmunol.177.3.1679 16849477

[101] Tan PH Yates JB Xue S-A Chan C Jordan WJ Harper JE. Creation of tolerogenic human dendritic cells via intracellular CTLA4: a novel strategy with potential in clinical immunosuppression. Blood (2005) 106:2936–43. 10.1182/blood-2005-05-1826 15994283

[102] Tuettenberg A Fondel S Steinbrink K Enk AH Jonuleit H. CD40 signalling induces IL-10-producing, tolerogenic dendritic cells. Exp Dermatol. (2010) 19:44–53. 10.1111/j.1600-0625.2009.00975.x 19889024

[103] Fleming BD Mosser DM. Regulatory macrophages: setting the threshold for therapy. Eur J Immunol. (2011) 41:2498–502. 10.1002/eji.201141717 21952805 PMC4299459

[104] Hutchinson JA Riquelme P Geissler EK Fändrich F. Human regulatory macrophages. Methods Mol Biol. 2011) 677:181–92. 10.1007/978-1-60761-869-0_13 20941611

[105] Riquelme P Haarer J Kammler A Walter L Tomiuk S Ahrens N. TIGIT + iTregs elicited by human regulatory macrophages control T cell immunity. Nat Commun. (2018) 9:2858. 10.1038/s41467-018-05167-8 30030423 PMC6054648

[106] Akagawa KS. Functional heterogeneity of colony-stimulating factor-induced human monocyte-derived macrophages. Int J Hematol. (2002) 76:27–34. 10.1111/j.1440-1843.2006.00805.x 12138892

[107] Li G Kim Y-J Broxmeyer HE. Macrophage colony-stimulating factor drives cord blood monocyte differentiation into IL-10(high)IL-12absent dendritic cells with tolerogenic potential. J Immunol. (2005) 174:4706–17. 10.4049/jimmunol.174.8.4706 15814695

[108] Lucas M Zhang X Prasanna V Mosser DM. ERK activation following macrophage FcgammaR ligation leads to chromatin modifications at the IL-10 locus. J Immunol. (2005) 175:469–77. 10.4049/jimmunol.175.1.469 15972681

[109] Zhang X Edwards JP Mosser DM. Dynamic and transient remodeling of the macrophage IL-10 promoter during transcription. J Immunol. (2006) 177:1282–8. 10.4049/jimmunol.177.2.1282 16818788 PMC2643023

[110] Verreck FA de Boer T Langenberg DM van der Zanden L Ottenhoff TH. Phenotypic and functional profiling of human proinflammatory type-1 and anti-inflammatory type-2 macrophages in response to microbial antigens and IFN-gamma- and CD40L-mediated costimulation. J Leukoc Biol. (2006) 79:285–93. 10.1189/jlb.0105015 16330536

[111] Hutchinson JA Ahrens N Geissler EK. MITAP-compliant characterization of human regulatory macrophages. Transpl Int Off J Eur Soc Organ Transplant. (2017) 30:765–75. 10.1111/tri.12988 28543878

[112] Napoli JL. Physiological insights into all-trans-retinoic acid biosynthesis. Biochim Biophys Acta (2012) 1821:152–67. 10.1016/j.bbalip.2011.05.004 21621639 PMC3179567

